# Skin Diseases and Syphilis

**Published:** 1896-10

**Authors:** 


### *SELECTIONS AND ABSTRACTS.*

### SKIN DISEASES AND SYPHILIS.

EDITED BY DR. M. B. HUTCHINS.

### TREATMENT OF SYPHILIS.

Dr. John Lindsay, of Philadelphia, read a paper upon this subject before the Alumni Association of Jefferson Medical College (September *Retrospect of Medicine and Pharmacy*, from *Therapeutic Gazette.)*

He justly says that no acid nor caustic application should be made to a sore on the penis because the inflammation thus produced may lead to error in diagnosis, or, if it be a true chancre, you do harm. (Cauterization is proper and beneficial in the case of a chancroid, if you can be positive that it is a chancroid; whereas it is not only useless, but positively injurious in the case of a chancre.) He advises cleanliness of the sore and a simple dressing, as with iodoform, calomel, aristol, etc. (For herpes a drying, soothing powder is sufficient; for chancroid, calomel, or calomel and bismuth; for a chancere, ung. hydrarg., diluted with zinc oxide ointment as required, acts excellently.)

He rightly advises that no specific treatment should be given until the appearance of secondary symptoms, and does not consider the diagnosis positive until these appear. (This is a good, safe rule and no time is lost by waiting, meanwhile getting the patient in the best possible condition. There are cases where the penile sore and the glandular enlargements are so typical that it is not necessary to wait for a skin eruption—which may never be clearly visible if it is a black negro—but these exceptions only prove the rule.)

He prefers giving the protiodide of mercury, because of its convenience and the patient's tolerance of it. He advises the gradual increase of the drug to the point of tolerance—to the physiological dose. Cut the dose then in half—the tonic dose—and keep the patient on it for eighteen months. (Of course the quantity given and the length of time it is continued will depend upon the condition of the patient, but his plan is good for a case which yields nicely.) Smoking, and a diet which may produce diarrhea, must be prohibited. (Chewing tobacco, the use of condiments, etc., also, injure the mouth.) After eighteen months he gives the patient a rest of a month and then begins "mixed treatment." He wisely says, do not use the bichloride with the iodide of potash, but always the biniodide of mercury, because you get in a mixture of bichloride of mercury and iodide of potassium an uncertain quantity of a newly formed biniodide of mercury. (Many prescribe the bichloride with the iodides, knowing that the biniodide will be formed, but it is better to have the biniodide to begin with for accurate dosage.) He advises the filling of the mixed prescription with compound tincture of gentian, rather than with syrup and water, to prevent gatric disturbance.

An intermittent treatment with the "mixed" is kept up, then, for six to nine months.

In the third year of the disease he gives iodide of potassium off and on. He thinks the patient pretty safe if no symptoms appear during the fourth year.

A simple, yet to many physicians unknown fact, he states in saying that mercury is the mainstay in the treatment both of early and late syphilis. Iodide of potassium is of value in the late stages, but chiefly assists in dispelling symptoms. (Mercury is the curative agent in syphilis, iodide of potassium simply removes symptoms. Antipyretics remove fever, they do not cure it—as witness typhoid.) He does not think it safe for a syphilitic to marry under three years after infection, better after four or five years, and only when the patient has been thoroughly and successfully treated with mercury.

The hypodermic use of mercury is desirable only in cases where the patient must be rapidly brought under its influence. (Of course no one should attempt the hypodermic method without a thorough knowledge of its technique.)

---

Drs. L. A. Stimson, A. G. Gerster, and B. F. Curtis, at a recent meeting of the New York Surgical Society, submitted the following report upon the use of erysipelas toxins in the treatment of malignant disease: "We believe that in the instances of apparent cure or marked improvement the correctness of the diagnosis is open to doubt. We, therefore, submit: 1. That the danger to the patient from this treatment is great. 2. Moreover, that the alleged successes are so few and doubtful in character that the most that can be fairly alleged for the treatment by toxins is that it may offer a very slight chance of amelioration. 3. That valuable time has often been lost in operable cases by postponing operation for the sake of giving the method of treatment a trial. 4. Finally, and most important, that if the method is to be resorted to at all, it should be confined to the absolutely inoperable cases."—*University Medical Magazine*, September. *Journal American Medical Association*, September 19, 1896.

---

Following are some tolerably well known prescriptions, reproduced from the *Monthly Retrospect* for September, 1896:

### LOCAL TREATMENT OF BALDNESS.

Tinct. cantharidis...4 fl. drs.

Tinct. capsici.......1 fl. oz.

Ol. ricini..½ fl. dr. to 1 fl. dr.

Alcoholis, q.s. ad....4 fl. ozs.

M. Sig—Rub thoroughly into the scalp.

### FEVER BLISTERS.

Camphor ............. 5 grs.
Arrowroot, powdered ..30 grs.
Bismuth subnitrate....30 grs.
Cold cream............ 4 drs.

M.

### FOR CORNS.

Painted on the corn night and morning for several days, when the corn will, as a rule, come readily away:—

Acid. salicylici......30 grs.
Ex. cannabis ind....10 grs.
Collodii...........4 fl. drs.

M.

### PATHOGENIC GERMS IN THE SCALP.

Besides the germs to which "dandruff" and its products are due, it is known that the staphylococci also find lodgment in the scales of the scalp. This being true it is the duty of the "aseptic" or "antiseptic" operator, either to have his scalp in a perfectly clean and healthy condition, or to wear a close cap when operating. A few loose scales shaken from the hair could nullify all the usual surgical precautions. This suggests the fact that there is no part of the body which receives as little cleansing as the scalp. Many scalps only need soap and water, but a great number have reached that stage where they need thorough and proper medicinal treatment.

